# Regional environmental controllers influence continental scale soil carbon stocks and future carbon dynamics

**DOI:** 10.1038/s41598-021-85992-y

**Published:** 2021-03-19

**Authors:** Daniel Ruiz Potma Gonçalves, Umakant Mishra, Skye Wills, Sagar Gautam

**Affiliations:** 1 Department of Fitotechnics and Plant Health, Universidade Estadual de Ponta Grossa, 4748, General Carlos Cavalcanti Avenue, Ponta Grossa, Paraná 84030-900 Brazil; 2 Bioscience Division, Sandia National Laboratory, Livermore, CA 94550 USA; 3 National Soil Survey Center, USDA- Natural Resource Conservation Center, 100 Centennial Mall North, Lincoln, NE 68508 USA

**Keywords:** Biogeochemistry, Carbon cycle

## Abstract

Understanding the influence of environmental factors on soil organic carbon (SOC) is critical for quantifying and reducing the uncertainty in carbon climate feedback projections under changing environmental conditions. We explored the effect of climatic variables, land cover types, topographic attributes, soil types and bedrock geology on SOC stocks of top 1 m depth across conterminous United States (US) ecoregions. Using 4559 soil profile observations and high-resolution data of environmental factors, we identified dominant environmental controllers of SOC stocks in 21 US ecoregions using geographically weighted regression. We used projected climatic data of SSP126 and SSP585 scenarios from GFDL-ESM 4 Earth System Model of Coupled Model Intercomparison Project phase 6 to predict SOC stock changes across continental US between 2030 and 2100. Both baseline and predicted changes in SOC stocks were compared with SOC stocks represented in GFDL-ESM4 projections. Among 56 environmental predictors, we found 12 as dominant controllers across all ecoregions. The adjusted geospatial model with the 12 environmental controllers showed an R^2^ of 0.48 in testing dataset. Higher precipitation and lower temperatures were associated with higher levels of SOC stocks in majority of ecoregions. Changes in land cover types (vegetation properties) was important in drier ecosystem as North American deserts, whereas soil types and topography were more important in American prairies. Wetlands of the Everglades was highly sensitive to projected temperature changes. The SOC stocks did not change under SSP126 until 2100, however SOC stocks decreased up to 21% under SSP585. Our results, based on environmental controllers of SOC stocks, help to predict impacts of changing environmental conditions on SOC stocks more reliably and may reduce uncertainties found in both, geospatial and Earth System Models. In addition, the description of different environmental controllers for US ecoregions can help to describe the scope and importance of global and local models.

## Introduction

Soils store the largest amount of carbon in terrestrial ecosystems containing around 1500 Pg C (Pg; 1 Pg = 10^15^ g) soil organic carbon (SOC) in top 1 m depth^1,2^. Understanding the relationship between SOC and its environmental controllers is key for accurately predicting climate and land use change impacts on SOC and reducing uncertainties in large scale carbon climate feedback projections.

Earth System Models (ESMs)^3^ are used to predict the global carbon climate feedbacks and simulate the future state of soils and ecology. Despite their key roles in determining the spatial heterogeneity of SOC and regulating the rate of SOC decomposition, many environmental factors that regulate soil formation are not adequately represented in current land surface models^4^. As a result, current land surface models poorly represent baseline SOC spatial heterogeneity^4,5^ and show large uncertainties in predicting future carbon climate feedbacks^6^. Burke, et al.^7^ reported that the quantity, spatial distribution, and decomposability of SOC stocks accounted for half of the overall uncertainty in predicting future carbon climate feedbacks and associated climate changes. Therefore, to reduce the uncertainty in future carbon climate feedback projections, it is critical to appropriately represent environmental controllers and the spatial heterogeneity of SOC in land surface models. One way to improve the spatial heterogeneity of SOC stocks in land surface models is to quantify and represent the environmental controls on SOC stocks consistent with field observations.

Statistical geospatial modeling can be used to quantify the heterogeneity of environmental controllers on SOC stocks from regional to national and global scales. Also, environmental controllers’ spatial gradient can be used in a space-for-time substitution approach to predict the spatiotemporal variation in SOC stocks^8^. O'Rourke, et al.^9^ stated that linking specific environmental controllers to SOC functions can be a way to toward more realistic projections in ESMs. Examples of this approach were applied for Australian^10^, Brazilian^11^ and other soils using geospatial modelling. The same way Weintraub, et al.^12^ highlighted the potential of soil observation networks to accelerate process representation in SOC models and improve our current capacity to make predictions.

In this study we used a database composed of 4559 soil profile observations in conterminous US and 56 environmental predictors representing climate, topography, soil type, geology, land use and water dynamics to fit a geographically weighted regression model. We used the model to predict and generate maps of current and future SOC in 21 US ecoregions. The maps were generated considering current and two shared socioeconomic pathways SSP126 and SSP585 from GFDL-EMS4 of Coupled Model Intercomparison Project phase 6 (CMIP6), corresponding of IPCC RCP 2.6 and 8.5 scenarios. To produce the SSP126 and SSP585 pathways maps, we used future temperature and precipitation regimes from GFDL-EMS4 predictions in conterminous US. Finally, the results were compared with total SOC stocks from GFDL-ESM4 model predictions.

## Results

**Figure 1:**
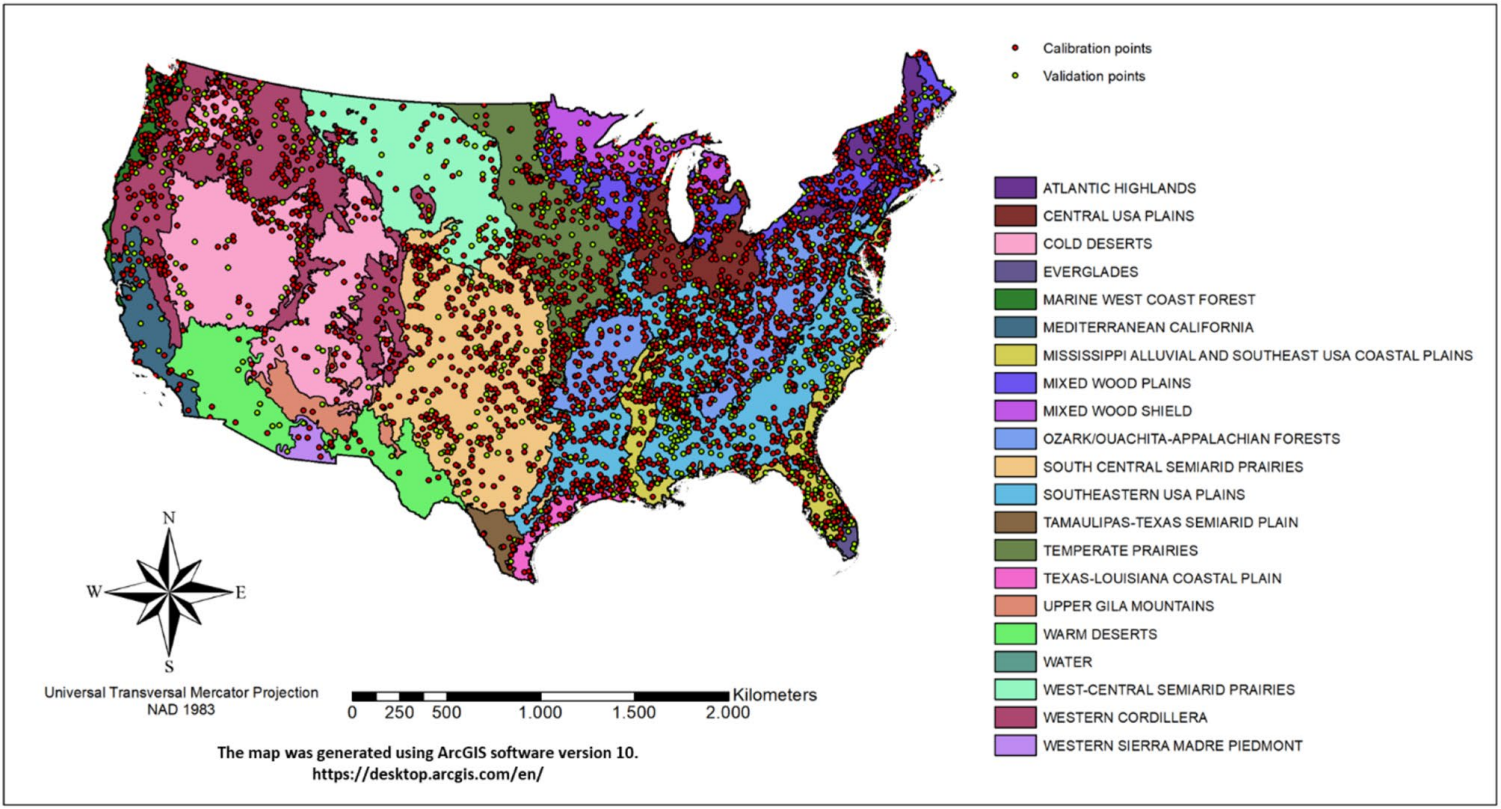
United States of America level II ecoregions, and the RaCA datapoints distribution, the red and green points were chosen for calibration and validation of the geographically weighted regression model respectively.

**Figure 2:**
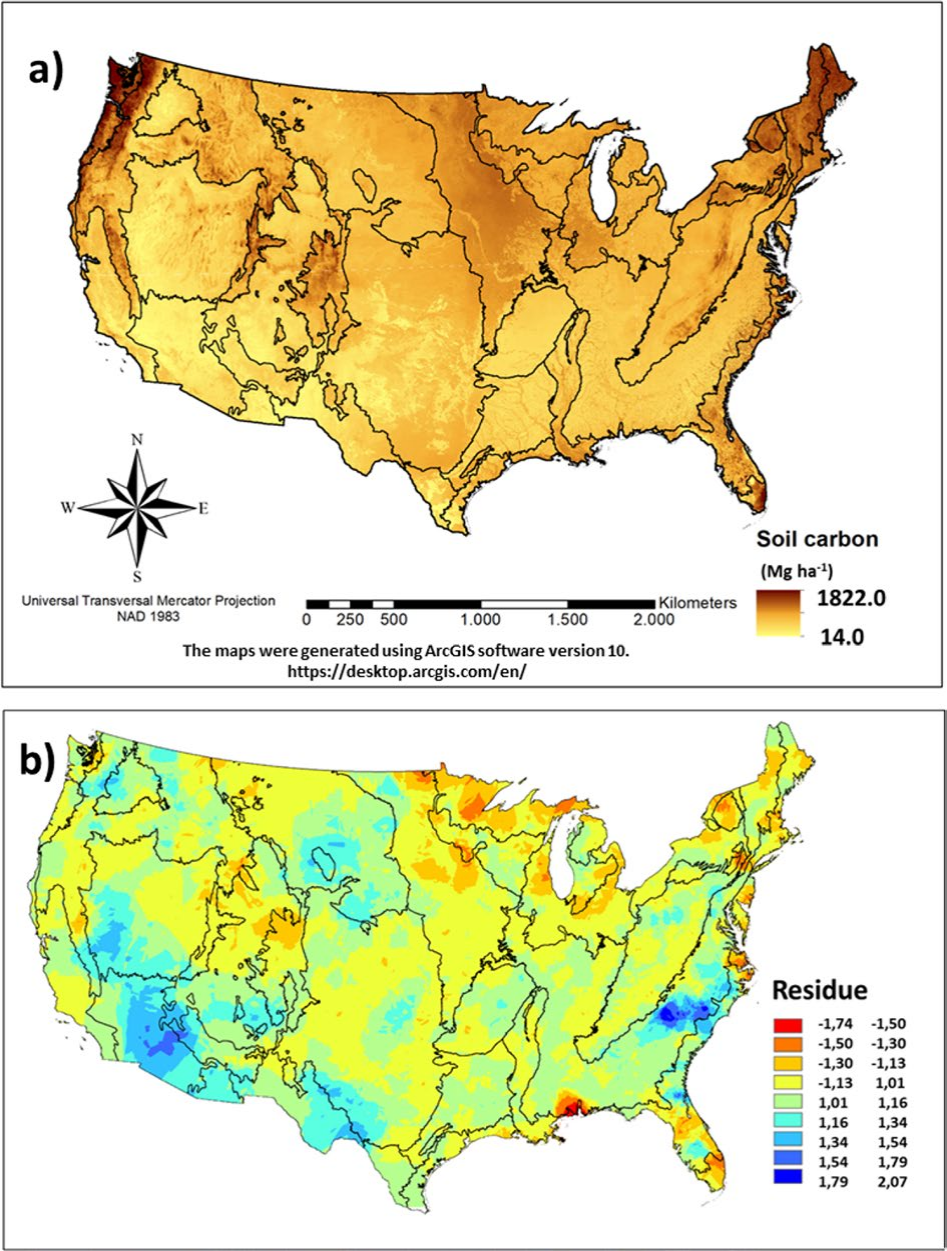
Maps of **s**oil organic carbon stocks distribution over continental United States of America obtained from the geographically weighted regression model (**a**) and model residuals (**b**). The black lines represent the borders of level II ecoregions described in Fig. 1.

The chosen model included 12 individual variables from the original 56 (Table 1) as yearly averages: Temperature, Precipitation, Atmosphere Net Radiation, Net Primary Productivity, (Normalized difference vegetation index) NDVI, Forest and Pastureland cover classes, Inceptisols, Vertisols, Ultisols, Spodosols, and Mollisols soil classes. The obtained SOC stocks map for all ecoregions (Figs. 1, 2) showed higher values in the north east and pacific north west, forested mountainous areas, south Florida, and central US prairies. The regions with highest mean SOC stocks were Everglades (777 Mg/ha), Mixed Wood Shield (660 Mg/ha), Mississippi Alluvial and Southeastern USA Plains (519 Mg/ha), Atlantic Highlands (456 Mg/ha) and Mixed Wood Plains (352 Mg/ha) (Table 2). The lowest values were found in Warm Deserts and Texas-Louisiana Coastal Plain, less than 65 Mg ha^−1^ C.

**Table 1:** Environmental predictors used for the geospatial modelling process.

Environmental predictor	Brief description	Data source	Resolution
**Climate predictors**			
Precipitation	30-yr (1981 to 2010) annual average precipitation	http://www.prism.oregonstate.edu/normals	800 m
Minimum temperature	30-yr (1981–2010) annual average minimum temperature	http://www.prism.oregonstate.edu/normals	800 m
Mean temperature	30-yr (1981–2010) annual average temperature	http://www.prism.oregonstate.edu/normals	800 m
Maximum temperature	30-yr (1981–2010) annual average maximum temperature	http://www.prism.oregonstate.edu/normals	800 m
Dew point temperature	30-yr (1981–2010) annual average dew point temperature	http://www.prism.oregonstate.edu/normals	800 m
Minimum vapor pressure deficit	30-yr (1981–2010) minimum vapor pressure deficit	http://www.prism.oregonstate.edu/normals	800 m
Maximum vapor pressure deficit	30-yr (1981–2010) maximum vapor pressure deficit	http://www.prism.oregonstate.edu/normals	800 m
Potential evapotranspiration	30-yr (1970–2000) potencial evapotransporation	https://cgiarcsi.community/	0.25 ^○^
Net radiation	2017 yearly average net radiation	https://neo.sci.gsfc.nasa.gov/	30 arc-seconds (≈ 1 km at equator)
**Land use and land cover predictors**			
Ecological region*	Ecological zone map at level II	https://www.epa.gov/eco-research/ecoregions-north-america	100 m
Net primary production	Annual terrestrial primary production	https://neo.sci.gsfc.nasa.gov/	0.25^○^
Normalized difference vegetation index (NDVI)	Annual Normalized difference vegetation index (Calculated as (NIR—RED)/(NIR + RED), where, NIR is near-infrared band)	https://neo.sci.gsfc.nasa.gov/	0.25^○^
National land cover database (6 classes)	Land cover of the United States for 2011	https://www.mrlc.gov/nlcd2011.php	30 m
**Topographic predictors**			
Elevation (DEM)	Land surface elevation	https://www.usgs.gov/core-science-systems/national-geospatial-program/	30 m
Aspect	Compass direction that the slope faces	Derived from DEM	30 m
Slope	Raise or fall of land surface	Derived from DEM	30 m
Plan curvature	Terrain curvature that is perpendicular to maximum slope direction	Derived from DEM	30 m
Profile curvature	Terrain curvature that is parallel to maximum slope direction	Derived from DEM	30 m
Total curvature	Combination of plan and profile curvature	Derived from DEM	30 m
**Soil and bedrock predictors**			
Soil orders (10 orders)	Taxonomy soil order	https://www.nrcs.usda.gov/wps/portal/nrcs/detail/soils/home/	100 m
Bedrock geology (23 classes)	Taxomony of bedrock geology	https://www.usgs.gov/products/maps/geologic-maps	1000 m

*The United States ecoregions were not used as environmental predictors, but for organize the various ecosystems prediction dynamics.

**Table 2:** Soil carbon stocks and model residue for US main ecoregions.

Ecoregion	Soil carbon stock (Mg ha^−1^)	Soil carbon stock (Tg)	Soil carbon stock SSP126 (Tg)	Soil carbon stock SSP585 (Tg)	Model residue (Mg ha^−1^)	Model residue (%)
Everglades	776.6	304.9	627.3	970.0	− 643.2	82.8
Mixed Wood Shield	661.1	2539.5	2603.5	2663.5	− 265.5	40.2
Mississippi Alluvial and Southeast USA Coastal Plains	519.3	3584.5	4220.1	4613.8	− 210.0	40.4
Atlantic Highlands	457.0	2949.8	2652.5	2405.5	− 132.4	29.0
Marine West Coast Forest	403.5	2101.9	2002.5	1939.6	− 104.7	25.9
Mixed Wood Plains	352.4	5266.9	5000.3	4772.7	− 151.1	42.9
Central Usa Plains	207.9	2481.3	2344.1	2241.3	− 74.2	35.7
Temperate Prairies	191.6	7289.2	7147.4	7012.0	− 21.9	11.4
Western Cordillera	170.5	9557.8	8751.5	8177.0	− 56.2	33.0
Texas-Louisiana Coastal Plain	163.7	631.2	633.4	625.2	− 41.2	25.2
Ozark/Ouachita-Appalachian Forests	123.5	4617.0	3881.1	3488.3	− 87.8	71.1
West-Central Semiarid Prairies	102.8	5637.8	5603.5	5566.6	− 7.3	7.1
Upper Gila Mountains	100.4	708.7	683.5	664.0	− 34.2	34.0
Cold Deserts	97.8	6927.5	6447.2	6106.8	− 27.4	28.0
Southeastern Usa Plains	97.5	8359.9	8115.0	7892.2	− 11.6	11.9
Mediterranean California	91.2	1134.4	1120.3	1129.2	− 22.9	25.1
South Central Semiarid Prairies	87.0	8184.3	7283.2	6703.1	− 24.7	28.4
Western Sierra Madre Piedmont	76.5	2339.7	2262.6	2215.0	− 6.1	8.0
Tamaulipas-Texas Semiarid Plain	61.7	368.9	332.5	300.0	− 2.0	3.3
Warm Deserts	48.0	223.1	244.9	264.0	0.6	1.2
Total (Pg)		75,208.2	71,956.2	69,749.7		

Predicted SOC stocks using geographically weighted regression (GWR) showed coefficient of variation (R^2^) of 0.48 (quasi-global). The GWR approach consistently underestimated the SOC stocks, bias is higher in the ecoregion with higher observed SOC stocks (Table 2). The bias was 83 and 71% in the Everglades and Ozark-Ouachita-Appalachian Forests; all other regions had bias less than 43%. Six Ecoregions presented model bias lower than 15. The model residual was not distributed homogeneously inside ecoregions, but some regions presented higher model bias than others (e.g., Mississippi delta and Warm Deserts SOC stocks were under and overestimated respectively) (Fig. 2).

**Figure 3:**
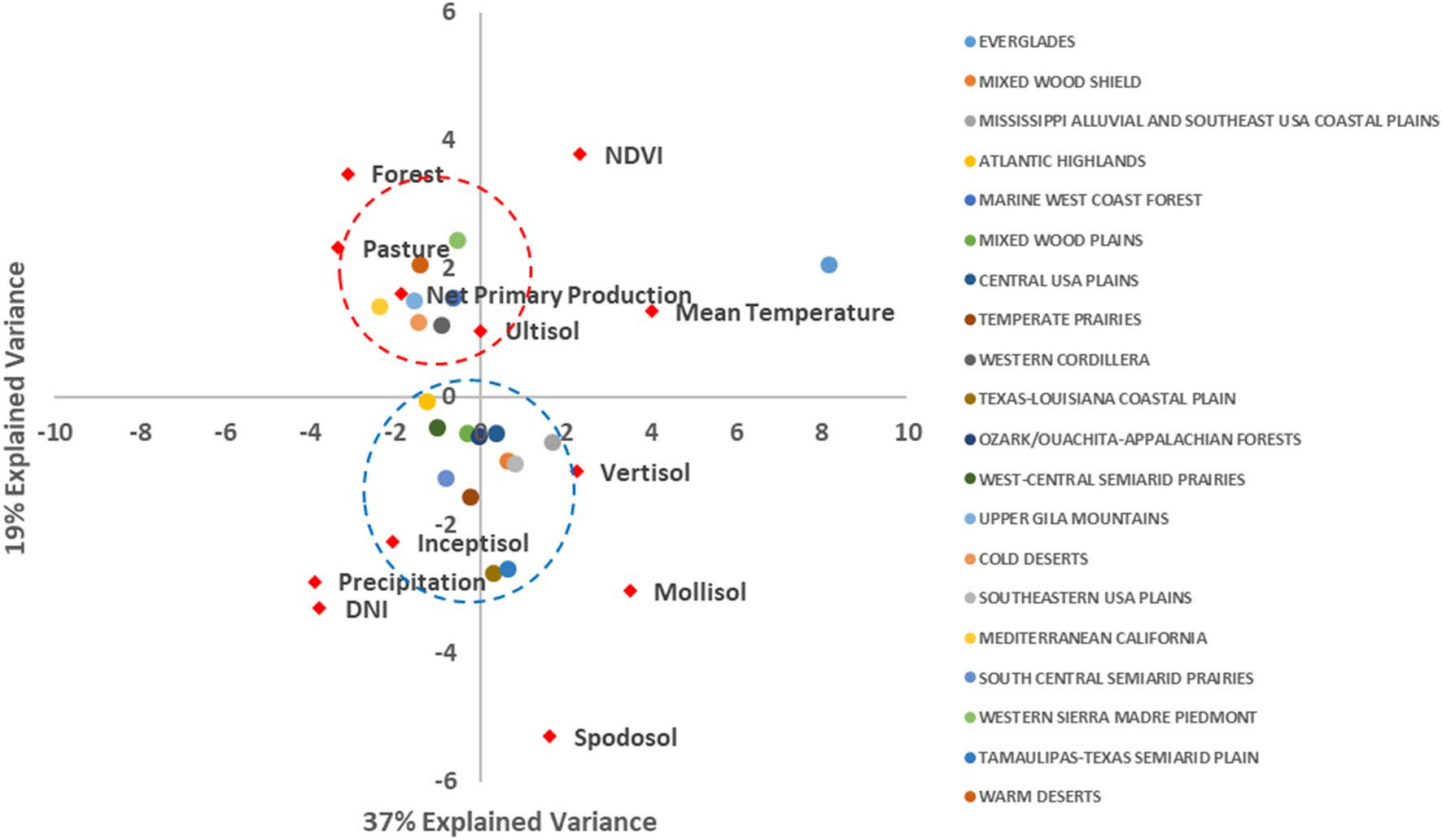
Principal component analysis relating the selected environmental predictors and the United States of America level II ecoregions.

SOC stocks were controlled by different environmental factors across US ecoregions (Fig. 3). The ecoregions, Mediterranean California, Cold Desert, Upper Gila Mountain, Warm Desert, Western Cordillera, and South-Central Semiarid Prairies were closely related to net primary production and vegetation types. In the Southern plains ecosystems, precipitation and net solar radiation were good predictors, and Everglades showed a different dynamics, presenting mean temperature and NDVI as good predictors. There was not a group of predictors highlighted for the other Ecoregions (Fig. 3). Mean temperature was positively related with SOC stocks in Everglades, Texas-Louisiana Costal Plains, Mediterranean California and Mississippi Alluvial and Southeastern USA Plains, on the other Ecoregions temperature increases resulted in reduction of SOC stocks. In Texas-Louisiana Coastal Plains and Mediterranean California SOC stocks were more sensitive to precipitation amount, which indicated it is a major diver of SOC stocks in warm and dry conditions. In the Everglades, Central USA Plains, Upper Gila Mountains and Mixed Wood Shield precipitation was negatively related on SOC stocks.

**Figure 4:**
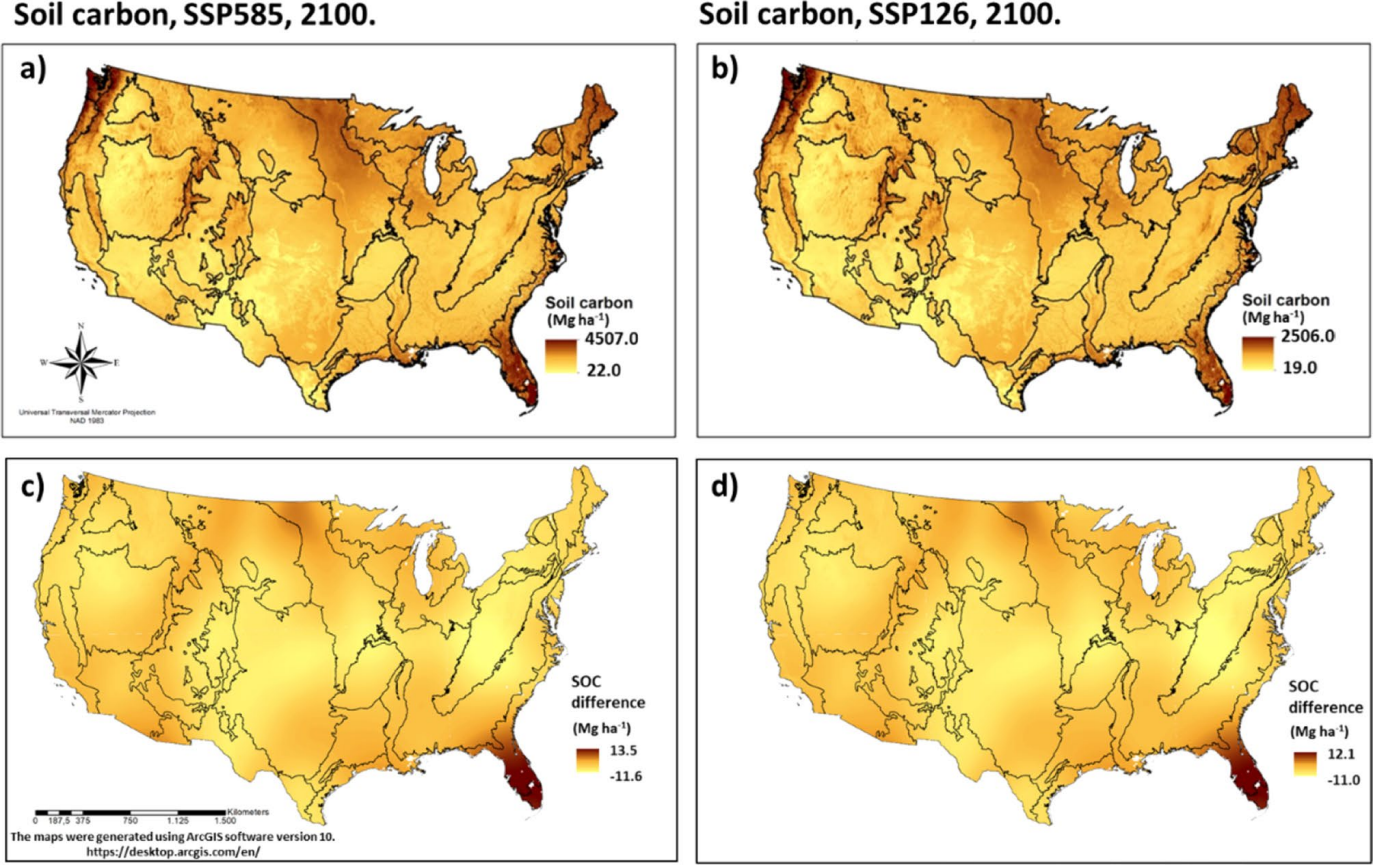
Soil organic carbon stocks prediction for 2100 in SSP585 (**a**) and SSP126 (**b)** scenarios using GFDL-ESM4 model of couple model intercomparison project phase 6 (CMIP6) climate projections data and the calculated difference compared with 2017 soil organic carbon stocks for SSP585 (**c**) and SSP126 (**d**). The black lines represent the borders of level II ecoregions described in Fig. 1.

**Figure 5:**
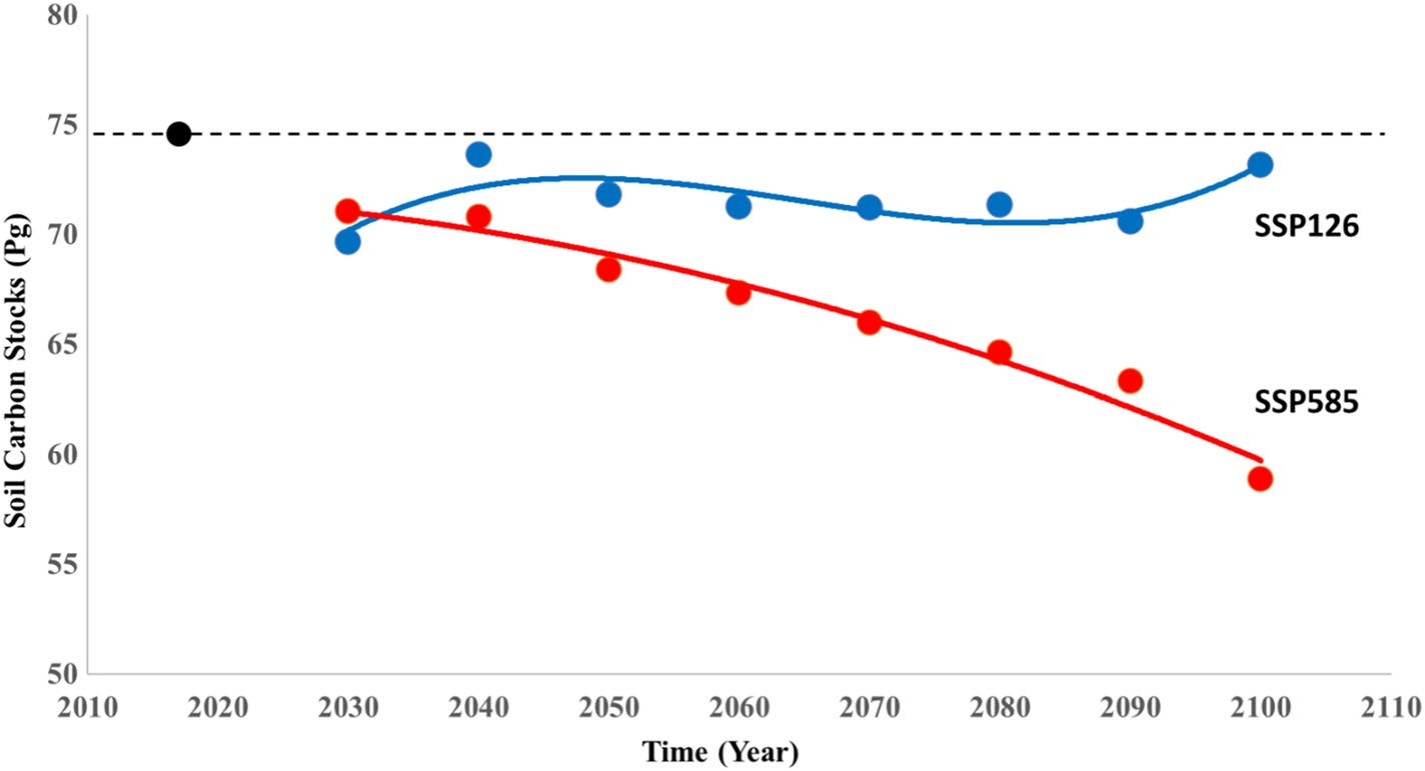
Soil organic carbon stocks predictions in continental United States of America for every ten years between 2020 and 2100 for SSP126 in blue and SSP585 in red.

The prediction for SSP126 and SSP585 scenarios using GFDL-ESM4 projection of temperature and precipitation showed a decrease in SOC stocks. The magnitude was about 4% to 7% (3252 and 5470 Tg) (Tg; 1 Tg = 10^12^ g) respectively (Table 2, Fig. 4). Both prediction maps (Fig. 4) showed an increase in SOC for northern prairies and Florida ecoregions. Other ecoregions showed decrease in SOC stocks indicating sensitivity of those ecosystems to future climatic changes. However, uncertainties related to sea level rise^13,14^, especially on SSP585 scenario, which predicts a raise between 60 and 110 cm util 2100, make it difficult to precisely determine SOC decreases in Florida Lowlands as this region is expected to lose land mass. Four ecoregions showed an increase in SOC stocks, Everglades, Mixed Wood Shield, Mississippi Alluvial and Southeastern USA Plains and Warm Deserts, it was most pronounced in the Everglades with a 192% increase in SOC. Model projections showed a reduction in SOC stocks for both lower and higher emission scenarios (SSP126 and SSP585) (Table 3, Fig. 5). The changes were not significant for SSP126 with approximately ~ 2% change. In contrast, for the SSP585 scenario, the reduction ranged between 5% in 2030 to 21% in 2100. Although the highest value of SOC observed in SSP585 (4507 Mg ha^−1^) was almost double the SSP126 (2506 Mg ha^−1^), total SOC stocks (the sum of all pixels in the maps) was lower (Fig. 4). We also compared our SOC results with the GFDL-ESM4 projections. Despite differences in the model’s resolution (800 m for geospatial model and 0.5° for GFDL-ESM4) and particularities of process representation (e.g., GFDL-ESM4 does not simulate for wetlands and peatlands); it also showed a decrease in SOC for SSP585 pathway, being more pronounced in northwest part of US (Supplementary Fig. 1). The lowest emission scenario (SSP126) maintained the current SOC stocks.

**Table 3:** Soil carbon stocks predictions for every 10 years between 2030 and 2100.

Year	SSP126				SSP585				Current scenario			
	Min	Max	Mean	Total C	Min	Max	Mean	Total C	Min	Max	Mean	Total C
	Soil C stocks (Mg/ha)			(Pg)	Soil C stocks (Mg/ha)			(Pg)	Soil C stocks (Mg/ha)			(Pg)
2017									0.2	24.3	96.3	74.5
2030	11.3	2895.3	90.0	69.6	11.3	4106.4	91.8	71.0				
2040	11.6	3920.2	95.1	73.6	11.4	4409.1	91.4	70.8				
2050	11.4	3074.9	92.8	71.8	11.3	8896.7	88.3	68.4				
2060	11.4	5626.1	92.0	71.2	11.2	6959.9	86.9	67.3				
2070	11.5	3776.5	92.0	71.2	11.4	6106.7	85.2	66.0				
2080	11.3	4445.1	92.1	71.3	11.4	11,526.6	83.4	64.6				
2090	11.4	2437.2	91.2	70.6	11.3	18,452.6	81.8	63.3				
2100	11.4	2149.4	94.5	73.2	11.3	13,047.4	76.0	58.8

## Discussion

Our baseline SOC stock distribution showed similar spatial distribution as the published RaCA map produced using an ordinary kriging spatial interpolation approach^15^. The high values of SOC stocks in coastal environments showed the high capacity of wetlands like the Everglades to store SOC. Wetlands potential for store SOC was also highlighted in other studies, Hinson, et al.^16^ estimated 1153–1359 Tg of SOC in the 0–100 cm in US peatlands, or about 19% of US SOC stocks. Similarly, the model bias in the SOC estimation for ecoregions with higher SOC stock were also documented in earlier studies^16^. Net primary production importance as predictor for SOC stocks in driest ecosystems like the North American deserts (Fig. 3) may be related to its sparse vegetation. Thereby, probably as the carbon input to soil is low compared to other ecosystems, the net primary production impact was captured as a limiting factor.

In this study, climatic variables were important predictors of SOC stocks in some ecoregions such as the Southeastern USA Plains and Everglades, showing the sensitivity of these ecosystems to projected climate change estimates. The higher influence of temperature and precipitation on SOC of wetlands indicate vulnerability of these system to future climate changes^17,18^. Although climate factor has been reported in this and other studies as important predictors for SOC stocks, it may be a proxy for geochemical factors that affect SOC directly like soil texture and microbial activity. Geochemical factors were also reported as important predictors for SOC stock in other studies^19^. Yang, et al.^20^ found soil moisture and texture as major drivers to explain SOC distribution in Tibetan plateau and Giardina, et al.^21^ suggested the change in soil respiration due to increased temperature and precipitation as major factor to determine the change in SOC stocks.

Some other factors used to predict SOC stocks are also expected to change in the future (e.g., the location of wetlands is expected to change with the change in hydrologic regime and wildfires may increase in some regions due to temperature raise and reduced precipitation). According to the fifth IPCC report^18^, the Southern US ecoregions are expected to get drier, opposite to the Everglades and the central region that are expected to experience higher precipitation. Although wetlands are expected to change due to increased CO_2_ concentration, precipitation change, frequent extreme events (e.g., floods, storms), sea level rise, frequent wildfires, increase methane emissions and temperature, the effect of climate change on SOC stocks is still not well known^22^. The complexity of the water cycle makes it difficult to capture all the feedbacks and peatlands/wetlands may emit more carbon than sequester^17^. It’s not clear whether climate change will increase or decrease wetland area over continental US, but the net primary production should increase^23^. Primary vegetation is expected to move South to North in continental US due to increase in temperature in higher latitude^23,24^. Isolated events like wildfires has increase in the last years in west conterminous US, resulting in tree mortality^25^. Although carbon emission from wildfires is certain, its effect is still not well understood because of vegetation regeneration^26^. On the other hand, US deserts may increase biomass production^18^.

The extreme SOC values showed no difference for record low between SSP126 and 585, but the record high was 41 to 500% higher in SSP585 compared to 126. Soil organic carbon stocks reduction accounting for conterminous US for SSP585 was highly pronounced compared to 126, highlighting SOC sensitivity to climatic factors. The main exception, Florida, showed an increase in SOC stocks (Table 2), that can be explained by the high sensitivity of this region to mean temperature (Fig. 3). Temperature and precipitation changing are expected to influence other factors such as vegetation and sea level, influencing SOC stocks. These changes, as well its effect over SOC stocks, takes time, since SOC stabilization in soils is a dynamic process that reaches a new equilibrium stage on ecosystems after years or even decades from perturbation. These factors can add uncertainties when using a spatial based approach instead of temporal^8^. Although, when comparing our results with GFDL-ESM4 model simulations that accounted for carbon climate feedbacks, the same pattern was observed, considering total SOC stocks reduction for SSP126 and SSP585.

Although SOC dynamics may be similarly affected by the explored predictors in all the studied region, our results indicated relative importance, since SOC stocks were limited for different predictors across different ecoregions. This way, changes in SOC stocks inside the same ecoregion was related to variation on the specific limiting factors. This is especially important for improve soil process representations in Earth System Models, where treat specific important factors more accurately in different ecoregions can be a strategy for improve models’ predictions. Higher precipitation and lower temperatures were associated with higher levels of SOC stocks in majority of ecoregions. Changes in land cover types (vegetation properties) was important in drier ecosystem as North American deserts, whereas soil types and topography were more important in American prairies. Wetlands of the Everglades was highly sensitive to projected temperature changes. Although represent these behaviors in Earth System Models can be a way to produce more accurate global predictions, interaction among factors can result in emergent complex interactions, difficult to derive especially when data is a limiting factor^12^. This shows the importance of local models as an alternative to produce more accurate predictions on its represented region. The development of appropriate observation networks may provide data for the development, benchmarking, and validation of statistical and process-based models, increasing our understanding of SOC cycle at different scales and improving the predictive accuracy^12,27^. In addition, the description of different environmental controllers for US ecoregions can help to describe the scope and importance of global and local models.

## Methods

### Study area and soil carbon profiles and observations

We performed this study in the continental US using 4559 georeferenced soil profile observations obtained from the Rapid Carbon Assessment^28^ project (Fig. 1). The soil profiles were distributed across the conterminous US covering 21 existing ecoregions described by the United States Environmental Protection Agency (EPA, https://www.epa.gov/eco-research/ecoregions)^29^, land cover and soil types. A total of 31,472 samples describe the soil profiles, 77% (24,192) were distributed between 0 and 100 cm, which was used for this study. Thus, approximately five samples per soil profile were used for calculating soil carbon stocks at 0–100 cm depth. SOC was measured by dry combustion and bulk density was modelled^30^. For calculating SOC stocks for each pedon/profile, a fixed depth approach was used. A more complete description of the methods used to analyze the samples can be obtained in the Rapid Carbon Assessment: Methodology, Sampling and Summary^15^. The measured SOC ranged from 1.37 to 11,981.0 Mg C ha^−1^ and with a mean of 209.6 Mg C ha^−1^. The lowest stocks were measured in desert biomes and highest in wetlands. The SOC stocks showed a unimodal positively skewed distribution to normalize the data for the modelling activities we applied a box-cox transformation.

### Environmental variables

Soil orders were obtained from the conterminous United States digital soil map derived from the gridded Soil Survey Geographic Database (gSSURGO) in 30 m resolution (https://data.nal.usda.gov/dataset/gridded-soil-survey-geographic-database-gssurgo) and aggregated into 10 soil orders: Alfisols, Andisols, Aridisols, Entisols, Histosols, Inceptisols, Mollisols, Spodosols, Ultisols, Vertisols. We obtained the bedrock geology types information^31^ from the United States Geological Survey (https://www.usgs.gov/products/maps/geologic-maps) map with 1 km resolution and aggregated the main geological features into 23 groups.

We obtained 30 years (1981–2010) mean annual average data of precipitation, maximum, minimum and mean temperature, mean dew point temperature and minimum and maximum vapor pressure deficit at 800 m resolution from PRISM website (http://www.prism.oregonstate.edu/normals/). Monthly mean net radiation was derived from NASA (https://neo.sci.gsfc.nasa.gov/) with 0.25 degrees resolution and compiled into average annual net radiation datasets. The potential evapotranspiration maps were acquired from Consultative Group on International Agricultural Research—Consortium for Spatial Information (https://cgiarcsi.community/) with spatial resolution of 30 arc-seconds (≈ 1 km at equator). The mean annual NDVI and net primary productivity datasets were obtained from NASA Earth observations at 10 km resolution (https://neo.sci.gsfc.nasa.gov/). We obtained US land cover data of 30 m resolution from multi resolution land characteristics (https://www.mrlc.gov/nlcd2011.php) and aggregated land cover types into 6 major categories, briefly: Open Water and Perennial Ice/Snow were grouped as Water, Developed Open Space, Low, Medium and High Intensity were grouped as Urban, Deciduous, Evergreen and Mixed Forest were grouped as Forest, Woody and Emergent Herbaceous Wetlands were grouped as Wetlands and the other classes were Rock, Cropland, Grassland, Pasture and Scrub. The Water, Urban and Bare Rock Land cover types were excluded from the analysis. All the chosen categorical variables were treated as individual predictors (e.g., We considered Alfisols, Andisols, Forest and Croplands as four predictors).

We obtained a digital elevation model (DEM) with 30 m resolution^32^ from US Geological Survey Database (https://www.usgs.gov/core-science-systems/national-geospatial-program/) and derived the following terrain attributes: Elevation, aspect, slope, plan curvature, profile curvature and total curvature in GIS environment using ArcGIS v 10^33^. The environmental predictors used are described in Table 1 and the ecoregion used for group the observations in Fig. 1. All the environmental dataset raster maps were resampled to 800 m, the climatic data resolution, and the values were extracted at SOC sample points and used in further analysis.

### Data pre-processing and geospatial model fit

The SOC data in the same ecoregion that were outside of 1.5*IQR where removed as outliers. First, we used plots to identify non-linear relationships that could not be captured by linear modelling. Further, to avoid including unnecessary environmental predictors in the model we generated Pearson correlation coefficients for all numerical predictors paired in a correlation matrix. When the pair showed a high correlation coefficient (*r* > 0.70) one of the predictors was removed, we choose keeping the one which its effect over SOC dynamics is better known according to current theory. To measure the model fit, we divided the database into training (75%; 3419 samples) and testing (25%; 1140 samples) datasets. The training dataset was used to fit the model and testing set to measure the model prediction capacity. We treated the nominal/categorical variables as dummy variables (e.g., 0 or 1).

After pre-processing, we fitted multiple regression models and used three selection criteria to choose the optimum set of linear predictors, variable significance according to F test at *p* < 0.05, best subset^34^, and R^2^. Using the significant environmental predictors, we fitted a GWR model, the adaptative bandwidth was chosen based on Akaike Information Criterion minimization^35^. Briefly, GWR works as a multiple linear regression that fits unique parameters for every feature in the dataset. Consequently, the result is a model with the same predictors for SOC stocks and different coefficients (β values) for each local and ecoregion. The spatial variation of the coefficients can be used to explore local ecoregion specific controllers for SOC stocks. A more complete description of GWR approach can be found in Fotheringham book^36,37^. The model was then applied to the test dataset which had an R^2^ of 0.48. We then used the GWR model to generate a SOC stocks map in in Mg ha^−1^ ^33^. Using the coefficients adjusted for the predictor variables in the GWR model, we calculated a correlation matrix and performed a principal component analysis to visualize the relationship between environmental predictors and ecoregions.

### Future climate change scenarios

We investigated the changes in SOC stocks until 2100 under SSP126 and SSP585 climate change scenarios of CMIP6^18,38^. The increase in atmospheric CO_2_ is expected to increase temperature and change precipitation averages. We obtained maps of mean air temperature and precipitation from National Climate Assessment (https://nca2014.globalchange.gov/highlights/report-findings/future-climate) and GFDL-ESM4 models (NOAA, National Oceanic and Atmospheric Administration) for every 10 years from 2030 to 2100 and used the GWR model to explore the effect of these changes on SOC stocks. Briefly, all the model predictors were kept constant with exception of temperature and precipitation (obtained from GFDL-ESM4 predictions), then the model was run to produce SOC maps. The generated SOC stock maps were also compared with GFDL-ESM4 model SOC stock maps for current and future climate change scenarios (CMIP6, https://esgf-node.llnl.gov/projects/cmip6/). This model was chosen because produced better predictions of SOC stocks in American biomes compared to other Earth System Models^4^. The maps were subtracted (e.g., 2100 SOC map for SSP126 scenario—current SOC map) for generate SOC stocks difference maps and those were used for comparing the differences between scenarios. All modelling processes were performed using R v. 3.6.1^39^.

## Supplementary Information

Supplementary Information

## Data Availability

The data that support the findings is available on request and the R code used in this study is on GitHub (https://github.com/D9989/Geospmodel).
