# Supplementary material for: Regional environmental controllers influence continental scale soil carbon stocks and future carbon dynamics
Source: Sci Rep. 2021 Mar 19;11:6474. doi: 10.1038/s41598-021-85992-y (PMC7979933; doi:10.1038/s41598-021-85992-y)
Supplement: Supplementary file 1 — Supplementary Information [file 41598_2021_85992_MOESM1_ESM.docx]

**Regional Environmental controllers influence continental scale soil carbon stocks and future carbon dynamics**

Daniel Ruiz Potma Gonçalves^a^*, Umakant Mishra^b^, Skye Wills^c^, Sagar Gautam^b^

^a^Universidade Estadual de Ponta Grossa, Department of Fitotechnics and Plant Health, 4748, General Carlos Cavalcanti Avenue, Ponta Grossa, Paraná, 84030-900, Brazil.

^b^Sandia National Laboratory, Bioscience Division, Livermore, CA 94550, USA.

^c^National Soil Survey Center, USDA- Natural Resource Conservation Center, 100 Centennial Mall North, Lincoln, NE 68508, USA.

*Corresponding author. Phone: +55 (42) 98425-4463, E-mail: drpgoncalves@uepg.br (Gonçalves, D.R.P), ORCID: http://orcid.org/0000-0003-2015-048X

Authors ORCID: Umakant Mishra, https://orcid.org/0000-0001-5123-2803; Skye Wills, https://orcid.org/0000-0001-5664-7733; Sagar Gautam, https://orcid.org/0000-0002-0828-1631

**Supplementary Table 1 –** Beta (β) values assigned during geographically weighed regression modelling for the environmental predictors using log soil carbon stocks as dependent variable.

| **Ecoregion** | **Soil carbon**  **(Mg ha^-1^)** | **Intercept** | **SD** | **Mean Temperature** | **SD** | **Precipitation** | **SD** | **Net Radiation** | **SD** | **Net Primary Production** | **SD** | **NDVI** | **SD** | **Forest** | **SD** |
| --- | --- | --- | --- | --- | --- | --- | --- | --- | --- | --- | --- | --- | --- | --- | --- |
| **Everglades** | **776.6** | 7.449063 | 0.043468 | 0.098463 | 0.006911 | -0.000836 | 0.000091 | -0.000763 | 0.000014 | -0.000069 | 0.000002 | 0.001868 | 0.000042 | -0.068702 | 0.005956 |
| **Mixed Wood Shield** | **661.1** | 5.585556 | 0.287053 | -0.014898 | 0.004081 | -0.000003 | 0.000097 | -0.000012 | 0.000083 | 0.000006 | 0.000008 | -0.000301 | 0.000316 | -0.029359 | 0.014198 |
| **Mississippi Alluvial and Southeast USA Coastal Plains** | **519.3** | 5.745040 | 1.113262 | 0.007892 | 0.034317 | 0.000094 | 0.000301 | -0.000201 | 0.000335 | -0.000023 | 0.000034 | 0.000800 | 0.001139 | -0.035559 | 0.044985 |
| **Atlantic Highlands** | **457** | 4.914008 | 0.086712 | -0.019370 | 0.010742 | 0.000240 | 0.000045 | 0.000123 | 0.000013 | -0.000007 | 0.000004 | 0.000414 | 0.000528 | 0.027009 | 0.009935 |
| **Marine West Coast Forest** | **403.5** | 5.621550 | 0.130892 | -0.017308 | 0.010723 | 0.000081 | 0.000052 | -0.000079 | 0.000027 | -0.000012 | 0.000015 | 0.001039 | 0.000838 | 0.218671 | 0.022450 |
| **Mixed Wood Plains** | **352.4** | 5.342803 | 0.401614 | -0.021411 | 0.009710 | 0.000108 | 0.000158 | 0.000038 | 0.000084 | -0.000002 | 0.000009 | 0.000127 | 0.000645 | 0.006533 | 0.031850 |
| **Central USA Plains** | **207.9** | 5.676704 | 0.172678 | -0.025675 | 0.009667 | -0.000069 | 0.000091 | -0.000019 | 0.000036 | -0.000005 | 0.000008 | 0.000375 | 0.000645 | 0.012645 | 0.028357 |
| **Temperate Prairies** | **191.6** | 5.761894 | 0.289033 | -0.021387 | 0.006738 | 0.000151 | 0.000103 | -0.000080 | 0.000079 | 0.000016 | 0.000006 | -0.000362 | 0.001030 | 0.007575 | 0.020867 |
| **Western Cordillera** | **170.5** | 5.569051 | 0.224150 | -0.023976 | 0.012922 | 0.000154 | 0.000114 | -0.000053 | 0.000033 | 0.000632 | 0.001513 | 0.000424 | 0.000782 | 0.098715 | 0.118255 |
| **Texas-Louisiana Coastal Plain** | **163.7** | 4.407586 | 0.281533 | 0.006538 | 0.003708 | 0.000275 | 0.000031 | 0.000086 | 0.000049 | 0.000016 | 0.000004 | -0.000606 | 0.000117 | 0.010187 | 0.011992 |
| **Ozark/Ouachita-Appalachian Forests** | **123.5** | 5.325406 | 0.272910 | -0.030207 | 0.010120 | 0.000101 | 0.000093 | 0.000030 | 0.000096 | -0.000014 | 0.000025 | 0.000766 | 0.001044 | 0.035386 | 0.015363 |
| **West-Central Semiarid Prairies** | **102.8** | 5.654390 | 0.228785 | -0.024175 | 0.005582 | 0.000236 | 0.000102 | -0.000079 | 0.000043 | 0.000090 | 0.000225 | 0.000721 | 0.000543 | 0.028399 | 0.067012 |
| **Upper Gila Mountains** | **100.3** | 6.055865 | 0.161412 | -0.015094 | 0.007356 | -0.000001 | 0.000092 | -0.000121 | 0.000035 | 0.000767 | 0.000825 | -0.000047 | 0.000778 | 0.156926 | 0.036178 |
| **Cold Deserts** | **97.8** | 5.581679 | 0.291469 | -0.015607 | 0.011893 | 0.000192 | 0.000131 | -0.000058 | 0.000045 | 0.000691 | 0.001128 | -0.000269 | 0.000738 | 0.126711 | 0.055822 |
| **Southeastern USA Plains** | **97.5** | 5.466392 | 0.527074 | -0.013018 | 0.015259 | 0.000126 | 0.000120 | -0.000068 | 0.000148 | -0.000014 | 0.000026 | 0.000554 | 0.001072 | 0.005998 | 0.032802 |
| **Mediterranean California** | **91.2** | 5.444300 | 0.109860 | 0.006347 | 0.001746 | 0.000272 | 0.000035 | -0.000069 | 0.000025 | 0.000736 | 0.001182 | -0.000762 | 0.000266 | 0.120378 | 0.052159 |
| **South Central Semiarid Prairies** | **87** | 5.570260 | 0.313219 | -0.021321 | 0.007204 | 0.000094 | 0.000186 | -0.000045 | 0.000044 | 0.000167 | 0.000505 | 0.000019 | 0.000754 | 0.120115 | 0.060898 |
| **Western Sierra Madre Piedmont** | **76.5** | 5.975552 | 0.102272 | -0.013701 | 0.002561 | 0.000011 | 0.000040 | -0.000117 | 0.000018 | -0.000573 | 0.000302 | 0.001354 | 0.000452 | 0.127297 | 0.008371 |
| **Tamaulipas-Texas Semiarid Plain** | **61.7** | 4.863718 | 0.068682 | -0.001492 | 0.001482 | 0.000191 | 0.000039 | 0.000017 | 0.000012 | 0.000005 | 0.000005 | 0.000019 | 0.000324 | 0.027118 | 0.014153 |
| **Warm Deserts** | **48** | 5.960922 | 0.251835 | -0.005936 | 0.010898 | 0.000042 | 0.000217 | -0.000128 | 0.000039 | 0.000988 | 0.001268 | 0.000006 | 0.001438 | 0.116149 | 0.060236 |
|  |  |  |  | **Pasture** | **SD** | **Inceptisol** | **SD** | **Mollisol** | **SD** | **Spodosols** | **SD** | **Ultisols** | **SD** | **Vertisols** | **SD** |
| **Everglades** | **776.6** |  |  | -0.226261 | 0.016588 | -0.043249 | 0.018602 | 0.228259 | 0.019893 | -0.040264 | 0.003329 | 0.106481 | 0.019165 | 0.285037 | 0.030102 |
| **Mixed Wood Shield** | **661.1** |  |  | 0.013154 | 0.026220 | 0.019838 | 0.058706 | 0.092714 | 0.009881 | -0.021687 | 0.009320 | 0.053211 | 0.089687 | 0.130950 | 0.036358 |
| **Mississippi Alluvial and Southeast USA Coastal Plains** | **519.3** |  |  | -0.060017 | 0.067945 | 0.020427 | 0.067699 | 0.207416 | 0.182206 | 0.056037 | 0.114973 | -0.029349 | 0.064757 | 0.043167 | 0.135836 |
| **Atlantic Highlands** | **457** |  |  | -0.052272 | 0.015248 | -0.016965 | 0.008640 | -0.054341 | 0.019058 | 0.006759 | 0.016044 | -0.072574 | 0.061494 | -0.175209 | 0.092977 |
| **Marine West Coast Forest** | **403.5** |  |  | 0.074507 | 0.027082 | 0.026143 | 0.011825 | -0.015066 | 0.024686 | 0.015017 | 0.065417 | 0.057281 | 0.046566 | 0.047061 | 0.103642 |
| **Mixed Wood Plains** | **352.4** |  |  | -0.007183 | 0.040217 | 0.013746 | 0.044892 | 0.025129 | 0.075039 | -0.013561 | 0.024422 | -0.009803 | 0.083884 | 0.004283 | 0.167577 |
| **Central USA Plains** | **207.9** |  |  | 0.045624 | 0.018971 | 0.071061 | 0.012556 | 0.088055 | 0.018923 | -0.053874 | 0.018009 | 0.052175 | 0.022304 | 0.167336 | 0.031485 |
| **Temperate Prairies** | **191.6** |  |  | 0.073953 | 0.017287 | 0.051050 | 0.037459 | 0.113927 | 0.021763 | -0.025027 | 0.044300 | -0.053702 | 0.063446 | 0.159277 | 0.022096 |
| **Western Cordillera** | **170.5** |  |  | 0.160731 | 0.061535 | 0.030344 | 0.077770 | 0.015823 | 0.032033 | -0.150218 | 0.159977 | 0.052857 | 0.126171 | 0.080554 | 0.111980 |
| **Texas-Louisiana Coastal Plain** | **163.7** |  |  | 0.020094 | 0.018508 | 0.039694 | 0.012280 | 0.176905 | 0.008247 | 0.219991 | 0.047948 | -0.058085 | 0.028439 | 0.166349 | 0.033851 |
| **Ozark/Ouachita-Appalachian Forests** | **123.5** |  |  | 0.014622 | 0.041389 | 0.030538 | 0.016895 | 0.054119 | 0.090241 | 0.106160 | 0.087316 | 0.002715 | 0.013826 | 0.112035 | 0.080011 |
| **West-Central Semiarid Prairies** | **102.8** |  |  | 0.163280 | 0.044637 | 0.097367 | 0.087396 | 0.052982 | 0.030488 | -0.133135 | 0.060607 | -0.111995 | 0.097107 | 0.081995 | 0.026843 |
| **Upper Gila Mountains** | **100.3** |  |  | 0.235820 | 0.030059 | 0.046524 | 0.051828 | 0.034573 | 0.021194 | -0.356178 | 0.199353 | 0.026655 | 0.225743 | 0.086061 | 0.199415 |
| **Cold Deserts** | **97.8** |  |  | 0.181473 | 0.052547 | 0.078786 | 0.058141 | 0.030180 | 0.022952 | -0.316903 | 0.228219 | 0.130107 | 0.118868 | 0.023623 | 0.133698 |
| **Southeastern USA Plains** | **97.5** |  |  | -0.003022 | 0.051725 | 0.032969 | 0.036654 | 0.127300 | 0.131384 | 0.116959 | 0.116718 | -0.018505 | 0.038326 | 0.100052 | 0.100377 |
| **Mediterranean California** | **91.2** |  |  | 0.068178 | 0.090741 | 0.072867 | 0.024779 | 0.014445 | 0.044202 | -0.472458 | 0.202733 | 0.122741 | 0.031283 | -0.183253 | 0.021052 |
| **South Central Semiarid Prairies** | **87** |  |  | 0.104937 | 0.058245 | 0.085938 | 0.058940 | 0.121673 | 0.031802 | 0.081397 | 0.213476 | -0.095330 | 0.064151 | 0.207611 | 0.071858 |
| **Western Sierra Madre Piedmont** | **76.5** |  |  | 0.289273 | 0.011552 | -0.027500 | 0.050435 | 0.003738 | 0.009067 | -0.426745 | 0.132438 | -0.201389 | 0.157044 | 0.111573 | 0.064660 |
| **Tamaulipas-Texas Semiarid Plain** | **61.7** |  |  | 0.035256 | 0.005107 | 0.051723 | 0.009191 | 0.182115 | 0.009738 | 0.308694 | 0.021634 | -0.103548 | 0.009728 | 0.213283 | 0.004854 |
| **Warm Deserts** | **48** |  |  | 0.186858 | 0.074379 | 0.047758 | 0.077968 | 0.022156 | 0.046325 | -0.354211 | 0.517678 | 0.057025 | 0.198061 | -0.039825 | 0.235878 |

*The main tree predictors are highlighted with colors (red = soil; dark red = vegetation; blue = climate); SD = Standard Deviation.

**Supplementary Table 2 –** Soil carbon metrics for all the studied scenarios.

| **Ecoregion** | **Area (Km²)** | **Current scenario (2017)** | | | |  | **SSP126 (2100)** | | | |  | **SSP585 (2100)** | | | |
| --- | --- | --- | --- | --- | --- | --- | --- | --- | --- | --- | --- | --- | --- | --- | --- |
|  |  | **Minimum (Mg ha^-1^)** | **Maximum (Mg ha^-1^)** | **Mean (Mg ha^-1^)** | **Sum (Tg)** |  | **Minimum (Mg ha^-1^)** | **Maximum (Mg ha^-1^)** | **Mean (Mg ha^-1^)** | **Sum (Tg)** |  | **Minimum (Mg ha^-1^)** | **Maximum (Mg ha^-1^)** | **Mean (Mg ha^-1^)** | **Sum (Tg)** |
| **Everglades** | 21680.8 | 20.5 | 1316.0 | 140.6 | 304.9 |  | 33.0 | 3419.9 | 289.3 | 627.3 |  | 45.5 | 6419.5 | 447.4 | 970.0 |
| **Mixed Wood Shield** | 210136.6 | 49.9 | 271.8 | 120.9 | 2539.5 |  | 48.9 | 253.1 | 123.9 | 2603.5 |  | 47.4 | 261.9 | 126.8 | 2663.5 |
| **Mississippi Alluvial and Southeast USA Coastal Plains** | 337128.3 | 15.1 | 1205.7 | 106.3 | 3584.5 |  | 21.1 | 2165.4 | 125.2 | 4220.1 |  | 26.9 | 3796.7 | 136.9 | 4613.8 |
| **Atlantic Highlands** | 150128.9 | 76.3 | 1526.4 | 196.5 | 2949.8 |  | 61.5 | 1468.4 | 176.7 | 2652.5 |  | 55.1 | 1369.8 | 160.2 | 2405.5 |
| **Marine West Coast Forest** | 82123.0 | 60.3 | 2156.1 | 256.0 | 2101.9 |  | 58.9 | 2419.5 | 243.8 | 2002.5 |  | 59.3 | 2761.4 | 236.2 | 1939.6 |
| **Mixed Wood Plains** | 388807.2 | 51.8 | 959.5 | 135.5 | 5266.9 |  | 49.9 | 963.4 | 128.6 | 5000.3 |  | 44.2 | 898.5 | 122.8 | 4772.7 |
| **Central USA Plains** | 224157.4 | 73.2 | 345.7 | 110.7 | 2481.3 |  | 60.3 | 352.6 | 104.6 | 2344.1 |  | 52.4 | 346.9 | 100.0 | 2241.3 |
| **Temperate Prairies** | 521081.8 | 37.0 | 300.4 | 139.9 | 7289.2 |  | 44.9 | 342.0 | 137.2 | 7147.4 |  | 41.1 | 410.5 | 134.6 | 7012.0 |
| **Western Cordillera** | 820602.7 | 41.6 | 2432.7 | 116.5 | 9557.8 |  | 37.2 | 2876.8 | 106.7 | 8751.5 |  | 34.4 | 3217.4 | 99.7 | 8177.0 |
| **Texas-Louisiana Coastal Plain** | 73357.1 | 40.1 | 212.0 | 86.1 | 631.2 |  | 35.7 | 260.8 | 86.3 | 633.4 |  | 31.9 | 299.1 | 85.2 | 625.2 |
| **Ozark/Ouachita-Appalachian Forests** | 520072.1 | 50.0 | 459.9 | 88.8 | 4617.0 |  | 46.9 | 373.2 | 74.6 | 3881.1 |  | 40.1 | 325.6 | 67.1 | 3488.3 |
| **West-Central Semiarid Prairies** | 590301.2 | 41.8 | 337.7 | 95.5 | 5637.8 |  | 45.8 | 305.7 | 94.9 | 5603.5 |  | 40.6 | 286.7 | 94.3 | 5566.6 |
| **Upper Gila Mountains** | 108760.6 | 36.8 | 152.5 | 65.2 | 708.7 |  | 31.1 | 153.9 | 62.9 | 683.5 |  | 27.8 | 163.6 | 61.1 | 664.0 |
| **Cold Deserts** | 1004801.9 | 38.5 | 361.0 | 68.9 | 6927.5 |  | 35.0 | 353.1 | 64.2 | 6447.2 |  | 32.6 | 366.8 | 60.8 | 6106.8 |
| **Southeastern USA Plains** | 1027615.6 | 28.3 | 353.5 | 81.4 | 8359.9 |  | 38.6 | 344.5 | 79.0 | 8115.0 |  | 39.5 | 459.1 | 76.8 | 7892.2 |
| **Mediterranean California** | 162253.5 | 23.1 | 327.0 | 69.9 | 1134.4 |  | 24.3 | 302.6 | 69.1 | 1120.3 |  | 24.9 | 302.2 | 69.6 | 1129.2 |
| **South Central Semiarid Prairies** | 995863.8 | 41.1 | 190.0 | 82.2 | 8184.3 |  | 33.8 | 172.6 | 73.1 | 7283.2 |  | 29.8 | 168.4 | 67.3 | 6703.1 |
| **Western Sierra Madre Piedmont** | 405925.4 | 28.0 | 224.5 | 57.6 | 2339.7 |  | 26.7 | 271.2 | 55.7 | 2262.6 |  | 24.3 | 319.0 | 54.6 | 2215.0 |
| **Tamaulipas-Texas Semiarid Plain** | 52603.9 | 37.7 | 126.2 | 70.1 | 368.9 |  | 34.2 | 112.4 | 63.2 | 332.5 |  | 31.1 | 102.8 | 57.0 | 300.0 |
| **Warm Deserts** | 42359.7 | 39.4 | 187.6 | 52.7 | 223.1 |  | 41.9 | 230.2 | 57.8 | 244.9 |  | 44.1 | 271.5 | 62.3 | 264.0 |
| **Total** |  |  |  |  | **75208.2** |  |  |  |  | **71956.2** |  |  |  |  | **69749.7** |

**Supplementary Table 3 –** Temperature and precipitation for SSP126 and SSP585 scenarios between 2030 and 2100 in conterminous US according to GFDL-ESM4 predictions.

| Scenario | Precipitation | 2030 | 2040 | 2050 | 2060 | 2070 | 2080 | 2090 | 2100 |
| --- | --- | --- | --- | --- | --- | --- | --- | --- | --- |
| SSP126 | Min | 217.5 | 260.8 | 230.1 | 198.7 | 191.8 | 240.5 | 218.8 | 236.4 |
|  | Max | 1626.6 | 1762.6 | 1762.8 | 1740.9 | 1809.9 | 1906.1 | 1724.9 | 1873.7 |
|  | Mean | 858.7 | 955.5 | 935.5 | 926.9 | 948.7 | 929.3 | 927.2 | 966.4 |
|  | Std | 355.3 | 382.6 | 393.6 | 377.0 | 406.5 | 412.9 | 366.2 | 408.2 |
| SSP585 | Min | 201.9 | 224.0 | 207.9 | 172.3 | 262.4 | 258.3 | 282.0 | 249.5 |
|  | Max | 1632.3 | 1653.3 | 1722.6 | 1889.9 | 1843.2 | 1784.8 | 1829.0 | 1679.5 |
|  | Mean | 869.0 | 902.3 | 883.2 | 909.8 | 939.7 | 947.4 | 968.6 | 897.8 |
|  | Std | 376.0 | 359.0 | 385.5 | 430.0 | 389.0 | 395.9 | 401.9 | 376.4 |
|  | Temperature |  |  |  |  |  |  |  |  |
| SSP126 | Min | 0.6 | 0.6 | 0.7 | 0.7 | 0.9 | 0.6 | 0.9 | 0.6 |
|  | Max | 23.4 | 23.8 | 23.7 | 24.0 | 24.1 | 24.1 | 23.9 | 23.9 |
|  | Mean | 12.0 | 11.7 | 12.0 | 12.1 | 12.2 | 12.1 | 12.1 | 12.0 |
|  | Std | 4.8 | 4.7 | 4.7 | 4.7 | 4.7 | 4.7 | 4.6 | 4.8 |
| SSP585 | Min | 0.4 | 0.4 | 1.1 | 1.3 | 2.1 | 2.7 | 3.4 | 3.8 |
|  | Max | 23.5 | 23.8 | 24.5 | 24.8 | 25.1 | 25.5 | 25.9 | 26.5 |
|  | Mean | 11.7 | 11.9 | 12.6 | 13.0 | 13.5 | 13.9 | 14.5 | 15.3 |
|  | Std | 4.8 | 4.8 | 4.9 | 4.8 | 4.7 | 4.5 | 4.6 | 4.7 |


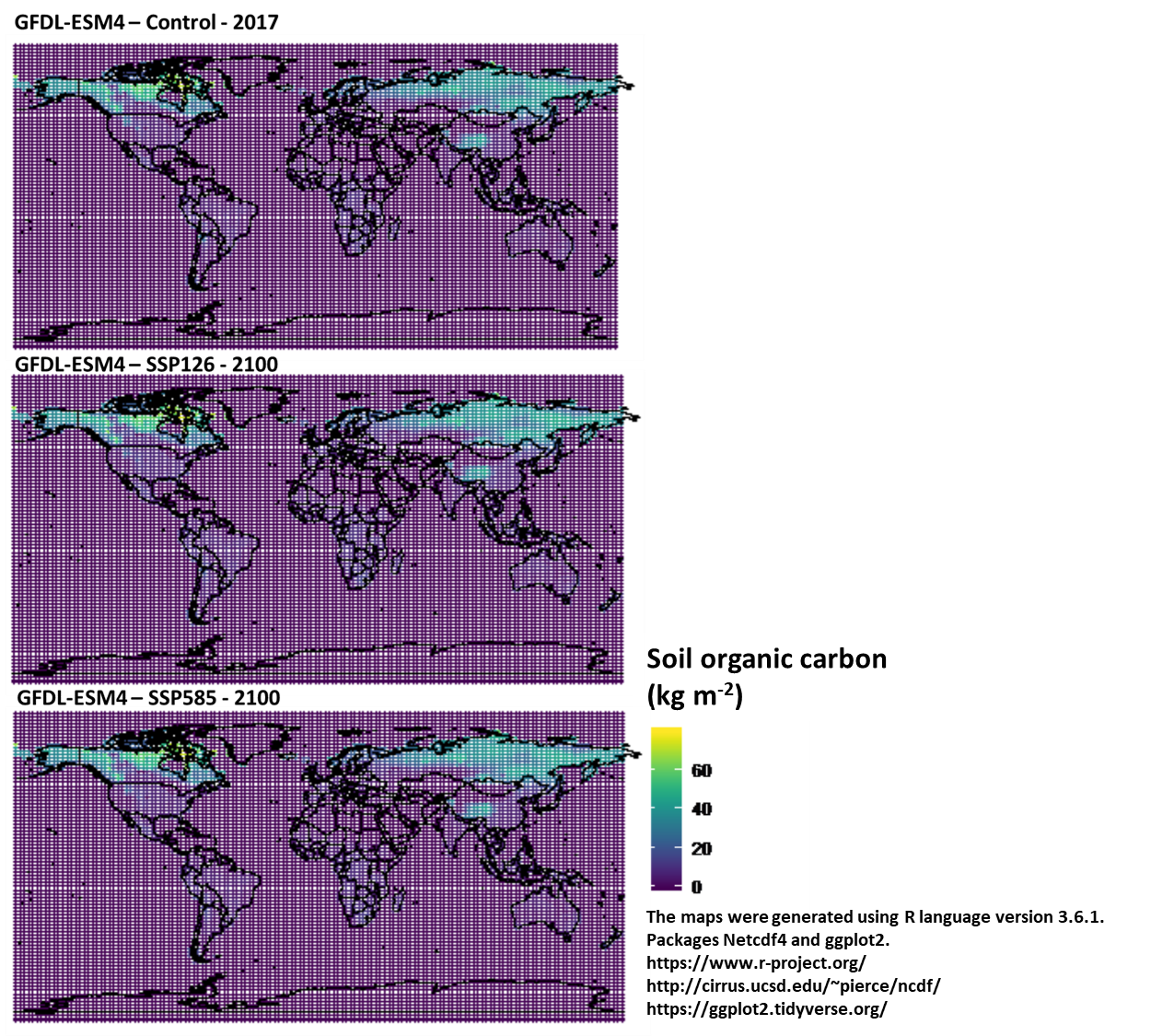


**Supplementary Figure 1:** Soil organic carbon stocks prediction for 2017 (current scenario) and 2100 for SSP126 and SSP585 using GFDL-ESM4 model.

*10 kg m^-2^ = 100 Mg ha^-1^.
